# Characterization of microbial metabolism of Syrah grape products in an in vitro colon model using targeted and non-targeted analytical approaches

**DOI:** 10.1007/s00394-012-0391-8

**Published:** 2012-06-16

**Authors:** Anna-Marja Aura, Ismo Mattila, Tuulia Hyötyläinen, Peddinti Gopalacharyulu, Veronique Cheynier, Jean-Marc Souquet, Magali Bes, Carine Le Bourvellec, Sylvain Guyot, Matej Orešič

**Affiliations:** 1 VTT Technical Research Centre of Finland, Tietotie 2, P.O.Box 1000, Espoo, 02044 Finland; 2 INRA, UMR 1083, Sciences Pour l’œnologie, Montpellier, France; 3 INRA, Unité Expérimentale de Pech Rouge, 11 430 Gruissan, France; 4 INRA, UMR 408 “Sécurité et Qualité des Produits d’Origine Végétale”, Avignon, France; 5 INRA UR117 Cidricoles et Biotransformation des Fruits et Légumes, Le Rheu, France

**Keywords:** Syrah grape, Red wine, Proanthocyanidins, In vitro colon conversions, Phenolic compounds, Short-chain fatty acids, Metabolite profiling

## Abstract

**Purpose:**

Syrah red grapes are used in the production of tannin-rich red wines. Tannins are high molecular weight molecules, proanthocyanidins (PAs), and poorly absorbed in the upper intestine. In this study, gut microbial metabolism of Syrah grape phenolic compounds was investigated.

**Methods:**

Syrah grape pericarp was subjected to an enzymatic in vitro digestion model, and red wine and grape skin PA fraction were prepared. Microbial conversion was screened using an in vitro colon model with faecal microbiota, by measurement of short-chain fatty acids by gas chromatography (GC) and microbial phenolic metabolites using GC with mass detection (GC–MS). Red wine metabolites were further profiled using two-dimensional GC mass spectrometry (GCxGC-TOFMS). In addition, the effect of PA structure and dose on conversion efficiency was investigated by GC–MS.

**Results:**

Red wine exhibited a higher degree of C1–C3 phenolic acid formation than PA fraction or grape pericarp powders. Hydroxyphenyl valeric acid (flavanols and PAs as precursors) and 3,5-dimethoxy-4-hydroxybenzoic acid (anthocyanin as a precursor) were identified from the red wine metabolite profile. In the absence of native grape pericarp or red wine matrix, the isolated PAs were found to be effective in the dose-dependent inhibition of microbial conversions and short-chain fatty acid formation.

**Conclusions:**

Metabolite profiling was complementary to targeted analysis. The identified metabolites had biological relevance, because the structures of the metabolites resembled fragments of their grape phenolic precursors or were in agreement with literature data.

**Electronic supplementary material:**

The online version of this article (doi:10.1007/s00394-012-0391-8) contains supplementary material, which is available to authorized users.

## Introduction

Syrah red grapes are used in the production of condensed tannin-rich red wine. In grape condensed tannins, also known as proanthocyanidins (PAs), are flavanol oligomers and polymers. They are extracted from grape skin and seeds into red wine in the winemaking process. In grapes, PAs are mainly composed of (−)-epicatechin, but also contain significant amounts of (+)-catechin, (−)-epigallocatechin and (−)-epicatechin 3-gallate subunits [1, 2]. Grape skin has a particularly high content of PAs, anthocyanins and phenolic acids, having a total content of polyphenols between 2.6 and 5.2 g/kg fresh berry weight depending on the variety [3], whereas flavonoids, (i.e. flavonols and dihydroflavonols) [4] and stilbenes [5] are present in lower amounts [6].

The content of polyphenols in red wine is affected by the grape matrix, extractability of the skin components [7] and their interaction with cell wall components [8] as well as by the winemaking process [9]. Flash détente is a particularly efficient process for increasing polyphenol extraction from grape skins [10]. Grape PAs with a low average degree of polymerization (aDPn) are well extracted into the wine in low alcohol levels, whereas those with high aDPn are not [3, 10–12]. The red wine polyphenols are therefore comprised of easily extractable, low aDPn PAs, anthocyanins, flavonols and phenolic acids and reaction products formed from them during winemaking and ageing [7].

PAs are poorly absorbed in the upper intestine [13], where they interact with macromolecules such as proteins, peptides and polysaccharides, or may inhibit enzymes [8, 14, 15]. Non-absorbed polyphenols are subjected to colonic microbiota. PAs, flavonoids, anthocyanins and phenolic acids undergo microbial metabolism to phenolic acid metabolites [16–20].

The intake of PAs is substantial among the polyphenols in human diets, and the average intake depends heavily on dietary habits. Average intake of PAs in the Mediterranean area by moderate (180 ml) red wine drinkers was approximately 100 mg PAs per day [21], and total intake of polyphenols in Finland was 863 ± 415 mg per day [22]. The intake of plant foods has been associated with lower incidence of chronic diseases [23]. The intake of PAs, hydroxycinnamic acids and anthocyanins, all present in grapes, can be associated with positive effects on hyperglycaemia, hyperlipidaemia, insulinaemia, insulin signalling and glucose uptake in adipose tissues, and prevention of detrimental effects on health related to metabolic syndrome, development of type 2-*diabetes mellitus* and obesity [24–29].

Colon microbiota has been associated with insulin sensitivity and regulation of fat storage [30, 31]. It is also probable that colonic metabolites, having a long residence time in the blood circulation [32–34], mediate the health benefits attributed to gut microbes. Novel experimental approaches such as comprehensive profiling of metabolites have recently been developed. These novel tools in systems biology afford systematic study of gut microbial metabolism depending on specific food components [19, 35, 36]. In this study, the in vitro colon model coupled with targeted and non-targeted analyses was applied to investigate the metabolism of different Syrah grape products in the presence of human faecal microbiota and to elucidate the effects of structure and dose of fruit PA fractions on the efficiency of microbial metabolism.

## Materials and methods

### Materials

Grape samples were prepared from *Vitis vinifera* var. Syrah grown at the Pech Rouge INRA experimental unit (Gruissan, France) and harvested in 2005 at commercial maturity. Syrah grape PA fraction and two apple PA fractions (*Malus domestica*, var. Marie Ménard (MM) and var. Avrolles (AV), 2005 season) were prepared. The apple and Syrah grape fractions were chosen because they contain high amounts of PAs with different average degrees of polymerization (aDPn) and galloylation.

Reagents for the targeted analysis of microbial metabolites of flavanols using gas chromatography with mass spectrometry (GC–MS) were as follows: heptadecanoic acid and succinic acid-2,2,3,3-d_4_ used as the internal standards were purchased from Sigma-Aldrich Inc., (St. Louis, USA). The following compounds were used as standards: benzoic acid (BA), 3-hydroxybenzoic acid (3-OHBA), 3-(4′-hydroxyphenyl) propionic acid (4-OHPPr) and 3-(3′,4′-dihydroxyphenyl) propionic acid (3,4-diOHPPr), which were purchased from Aldrich, (Steinheim, Germany); 4-hydroxybenzoic acid (4-OHBA), 2-(3′-hydroxyphenyl) acetic acid (3-OHPAc) and 2-(3′,4′-dihydroxyphenyl) acetic acid (3,4-diOHPAc), which were purchased from Sigma (St. Louis, USA); 3-phenylpropionic acid (3-PPr) and 3,4-dihydroxybenzoic acid (3,4-diOHBA), which were purchased from Fluka (Buchs, Switzerland); and 3-(3′-hydroxyphenyl) propionic acid (3-OHPPr) was purchased from Alfa Aesar (Karlsruhe, Germany). *N*-Methyl-*N*-trimethylsilyl-trifluoracetamide (MSTFA) from Pierce (Rockford, USA) was used as the derivatization reagent.

For comprehensive profiling of small polar metabolites using two-dimensional GC coupled to time-of-flight mass spectrometry (GCxGC-TOFMS; Leco, Inc., St. Joseph, MI), the internal standard was 2-hydroxycinnamic acid (mainly trans; Aldrich Inc. H2, 280-9; 97 %; St. Louis, USA), and additional standards of phenolic metabolites were 4-methylcatechol (Aldrich, Steinheim, Germany), vanillic acid (3-methoxy-4-hydroxybenzoic acid; Fluka, Buchs, Switzerland)), 4-hydroxycinnamic acid (Sigma, St. Louis, USA), gallic acid (Extrasynthése, Genay, France) and ferulic acid (Sigma-Aldrich, St Louis, USA). Prior to the addition of MSTFA, methoxyamine hydrochloride (2 %) in pyridine (MOX; Pierce, Rockford, USA) was used in the derivatization process for GCxGC-TOFMS.

### Preparation of grape samples

Three grape samples were processed. A crude grape pericarp powder was obtained from berries after removal of seeds, freezing in liquid nitrogen, grinding and freeze-drying. The pericarp powder contained skin and pulp. The skin PA fraction was prepared according to a procedure upscaled from that described by Souquet et al. [2]. The third sample was a de-alcoholized red wine made from the same Syrah grapes. The red wine was obtained by flash détente (a process known to increase PA content by about 20–30 % [10]) and fermentation of skins. After alcoholic and malolactic fermentation, de-alcoholization was performed by evaporation under reduced pressure in 45-l batches at a temperature below 40 °C. The red wine batches were pooled and stored at 4 °C under nitrogen atmosphere and then formulated (with glycerol, sugar, and aromas), filtered, bottled and pasteurized. Red wine was freeze-dried just before the colon model experiments. An unopened bottle of red wine was stored in cold (+4 °C) darkness for 3 years, after which it was freeze-dried prior to the experiment coupled with metabolite profiling.

### Preparation of apple samples

Purified PA extracts from apple corresponded to reversed-phase SPE extracts obtained from aqueous acetone extracts of apple powders as described by Guyot et al. [37]. Samples named as MM PA and AV PA were obtained from Marié Ménard and Avrolles varieties, respectively.

### Characterization of the fruit samples

Phenolic composition analysis of the grape material (before and after the in vitro digestion) and of the grape skin PA fraction was performed as described by Mane et al. [3]. Also, phenolics were extracted from the grape samples (before and after the in vitro digestion) as described by Mane et al. [3]. Simple phenolic compounds in the grape extract and wine were analysed by reversed-phase HPLC using Waters system (Milford, MA) equipped with a photodiode array detector (W2996). Samples (5 μl) were injected onto a reversed-phase Atlantis T3 column (5 μ, 250 mm × 2.1 mm) supplied by Waters, protected by a guard column of the same material and maintained at 38 °C. Elution was carried out with a gradient of acetonitrile/water/formic acid (80:15:5, v/v/v) in water/formic acid (95:5,v/v). Concentrations were calculated from peak areas at 520 nm for anthocyanins, at 360 nm for flavonols, at 320 nm for hydroxycinnamic acid derivatives and at 280 nm for flavan-3-ols and gallic acid. Malvidin 3-glucoside, quercetin 3-glucoside and caffeic acid were used as external standards for calibration of anthocyanins, flavonols and hydroxycinnamic acids, respectively.

Proanthocyanidins were analysed by reversed-phase HPLC after acid catalysed depolymerization in the presence of phloroglucinol, as previously described [3, 38]. The concentration of each unit released after phloroglucinolysis was calculated from its peak area at 280 nm (i.e. flavan-3-ols from terminal units and the corresponding phloroglucinol derivatives from extension and upper units), using the calibration curve established for the corresponding standard, either commercial ((+)-catechin, (−)-epicatechin, (−)-epigallocatechin and (−)-epicatechin-3-gallate) or purified in the laboratory (phloroglucinol derivatives). Eventual differences in the dilution or injection volumes were compensated for by taking into account the peak area of the internal standard (methylparaben). Total flavan-3-ol content was calculated by summing all units, and aDPn was calculated as the molar ratio of total released units to total terminal units. The percentages of epicatechin gallate units (% gallate) and of epigallocatcehin units (% epigallocatechin) were also calculated.

Red wine phenolics were analysed as described by Ducasse et al. [39]. For purified apple PA fractions, polyphenol analyses were performed by HPLC following thiolysis according to the procedure described by Guyot et al. [40].

Simple sugars (glucose, fructose and sucrose) were measured colorimetrically using the solutions and instructions from the Boehringer analysis kit (R-Biopharm, St Didier au Mont d’or, France). Alcohol-insoluble solids were isolated from grape powders (native and digested) by extensive washing of the powders until the extracts were sugar free, as described by Renard [41]. Red wine and PA fractions were directly submitted to polysaccharide analysis.

The individual neutral sugars were analysed by gas chromatography (capillary column of 30 m × 0.25 mm i.d. coated with DB225, 0.15 μm film thickness, J & W Scientific, Folsom, USA) at 215 °C, using hydrogen as carrier gas, after sulphuric acid hydrolysis (1 M, 3 h, 100 °C) and derivation to alditol acetates [42]. AIS preparations from grape powders were submitted to pre-hydrolysis in 13 M sulphuric acid (1 h, room temperature) [43]. Myo-inositol was used as internal standard. Uronic acids were determined spectrophotometrically by *m*-hydroxydiphenyl assay as described by Blumenkrantz and Asboe-Hansen [44] after acid hydrolysis of cell walls (Saeman procedure) with galacturonic acid as external standard.

### Enzymatic in vitro digestion

Enzymatic in vitro digestion of 1.5 g (d.w.) sample as described in Aura et al. [45] was scaled up sevenfold and performed under anaerobic conditions at 37 °C for the freeze-dried grape pericarp powder. The powder was slightly acidic, and thus, the mouth stage (neutral) was slightly acidic (pH 4.5), stomach was acidic (pH 2.4) and duodenum was neutral (pH 6.5). The conditions were mimicked by adding salivary α-amylase (mouth), hydrochloric acid and pepsin (stomach) and 150 mM sodium bicarbonate, pancreatin, bile and mucin (duodenum). Incubation was performed at 37 °C with magnetic stirring (250 rpm), and the digestion procedure was performed under anaerobic conditions to prevent oxidation of the components. Syrah grape solids were not apparent from the soluble part after digestion, and therefore, the entire sample was freeze-dried until analysis or application to the in vitro colon model.

### In vitro colon model

Freeze-dried Syrah grape powders or red wine were dosed 100 mg d.w./10 ml of faecal inoculum, whereas PA fractions were dosed 25 mg d.w./10 ml in the comparison with Syrah products and fruit PA fractions, and 28 mg, 14 mg and 7 mg/10 ml faecal inoculum in the experiment investigating the dose effect. Four colon model experiments were performed under strictly anaerobic conditions according to Aura et al. [18, 46], with the following specifications: Faecal suspensions were prepared for each experiment by pooling and suspending the faeces of at least 4 healthy donors to 0.11 M—carbonate—0.02 M phosphate buffer (pH 5.5) [47] in a Warring-Blender. The suspensions were filtered through a 1-mm sieve, diluted to 10 % (w/v) and applied immediately to the samples. Samples were incubated in a water bath at 37 °C for 0, 2, 4, 6, 8 and 24 h and stirred magnetically (250 rpm), unless otherwise stated.

In the experiments for targeted analysis by the GC–MS instrument, aliquots were drawn from the bottles and microbial metabolites and short-chain fatty acids (SCFA) were analysed after extraction of each aliquot. In the GCxGC-TOFMS experiment, aliquots were drawn from the bottles for SCFA analysis, and microbiota was removed using 0.2-μm PTFE filters (Millipore Corp., Bedford, MA, USA) before extraction of phenolic metabolites.

### Description of the study design

**Fig. 1:**
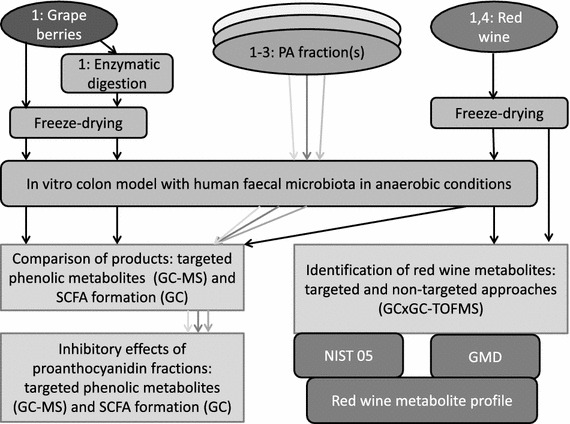
Description of the study design for four experiments using in vitro digestion models coupled with targeted and non-targeted analytical approaches

Syrah grape pericarp powder underwent the in vitro enzymatic digestion prior to the in vitro colon model, whereas red wine and all the PA fractions were applied to the colon model directly. The formations of microbial metabolites from the grape pericarp powders with or without enzymatic in vitro digestion, from the red wine and from the grape skin PA fraction were compared in the first experiment. The fruit PA fractions were compared in the second experiment investigating the effects of structural differences of PA fractions on microbial metabolism. The effect of dose of Syrah PA fraction on the microbial metabolism was investigated in the third experiment using three dose levels. A targeted GC–MS analytical approach was used in the three experiments. The red wine was chosen for further studies using a comprehensive non-targeted profiling of microbial metabolites by GCxGC-TOFMS in the fourth experiment. Short-chain fatty acid (SCFA) formation was also determined by gas chromatography as a reference for carbohydrate degradation. In all experiments, faecal suspension alone and in the fourth GCxGC-TOFMS experiment also red wine in buffer were used as controls. The description of the study design is shown in Fig. 1.

### Analysis of microbial metabolites

Total SCFA were analysed by gas chromatography with an FID detector after diethyl ether extraction according to Aura et al. [48]. The sum of SCFAs included the concentrations of acetic, propionic and butyric acids.

For targeted GC–MS analysis, microbial metabolites were extracted from faecal suspensions (1 mL) twice with 3 ml of ethyl acetate, and the solvent was evaporated. Derivatization was performed by adding dichloromethane (100 μl) and MSTFA (30 μl) and incubating for 5 min at 50 °C. The targeted analysis of microbial metabolites was performed by gas chromatography with mass detection (GC–MS) using selective-ion-monitoring (SIM) as described by Bazocco et al. [17] with authentic standards and heptadecanoic acid and succinic acid-2,2,3,3-d_4_ as internal standards. The metabolite formation was calculated as μmol/L of formed metabolite at each time point and expressed as averages and standard deviations.

For GCxGC-TOFMS, the derivatization was performed as follows: Internal standard (15 μL of 123 ppm 2-hydroxycinnamic acid) was added to 1 ml of filtered microbiota-free faecal water, and the samples were extracted twice with 2 ml of ethyl acetate. The extracts were evaporated to dryness under nitrogen and derivatized with 25 μl of MOX (1 h, 45 °C) and 25 μl of MSTFA (1 h, 45 °C). 5 μl of retention index standard mixture with five alkanes at 800 ppm was added to the metabolite mixture. In the targeted analysis, the metabolite formation was calculated as μmol/l of formed metabolite at each time point and expressed as averages and standard deviations. Analysis by GCxGC-TOFMS was performed as described in Aura et al. [49].

Measurement data from GCxGC-TOFMS were first processed by ChromaTOF software, which identifies compounds by matching deconvoluted spectra against an NIST05 mass spectral library. The results were exported to text files, and the in-house developed software Guineu [36] was used for aligning and normalization of compounds in different data sets for further analyses. The original GCxGC-TOFMS data include retention times, retention indices (RI), spectral information for possible identification, spectral similarity value (S = 0–1,000) and peak response data. The linear retention indices were calculated on the basis of the retention times of the compounds and the retention times of the retention index standards (*n*-alkanes). Alignment of the data was performed on the basis of retention indices, second-dimension retention times and spectra.

After alignment of the GCxGC-TOFMS data, two filtration criteria were utilized for preliminary identification: spectral match >850 and retention index (RI) as follows: RI_exp_-RI_lit_ < 25 or RI_exp_-RI_std, exp_ < 25, in which RI_exp_ was the experimental RI for a compound, RI_lit_ was the literature value for the identified compound and RI_std, exp_ was the experimental RI value for a standard compound. Compounds not fulfilling the criteria were renamed as *unknowns,* and relevant compounds were subjected to further identification. The identification of the *unkowns* at this stage was based on a spectral search from the NIST05 library or the in-house collected library and their retention indices.

### Statistical analysis

Two-way ANOVA for repeated measures was applied on quantitated metabolites by using a program designed for MatLab (R2008b). The program evaluated the responses against each substrate and the faecal control. Significant (*p* < 0.05) differences from the faecal control were designated as small letters, and different letters corresponded significantly (*p* < 0.05) to different levels of responses within a time point, unless otherwise stated.

Two-way ANOVA was also performed for non-targeted GCxGC-TOFMS data using the *aov* function of the *stats* package in *R* statistical programming language (http://www.r-project.org). The multiple hypothesis testing problem was addressed by correcting the *p* values to control the false discovery rate (FDR) using the *p. adjust* function of the *stats* package. Those metabolites showing FDR *q*-values lower than 0.0001 were included in the visualization by heat maps, which were used for displaying the relevant metabolites. Heat maps were produced with *R* using the *heatmap.2* function of the *gplots* package. Differences at each time point were evaluated by a two-sided *t* test at each time point using the *t* test function of the *stats* package. The asterisks shown in the heat map indicate significant differences in means at each time point based on the *t* test (*p < 0.05; **p < 0.01; ***p < 0.001).

GOLM Metabolome Database (GMD) (http://gmd.mpimp-golm.mpg.de/search.aspx) and the *Guineu* program [36] were utilized for second-stage identification of those compounds that lacked spectral matches with compounds from the NIST05 or in-house collected libraries. GMD database allows searching of the database based on submitted GC–MS spectra, retention indices and mass intensity ratios. In addition, the database allows a functional group prediction, which helped to characterize *unknown* metabolites without available reference mass spectra in the GMD.

The visualization was performed by calculating 2-based logarithmic fold changes of the relative peak areas from GCxGC-TOFMS analysis against the corresponding controls: faecal control (no red wine; Supplement Fig. 4A) or red wine in buffer (no faecal microbiota; Supplement Fig. 4B). The profile of the individual metabolite was visualized as colour intensities (red as over-expression and blue as under-expression) and the time point specific significances (*t* test *p* values) as asterisks against the corresponding control. The non-targeted metabolite profiling was semi-quantitative. The names of the over-expressed metabolites were verified by comparing the mass spectra with those found in GMD and the names for the *unkowns* were named according to the group specifications and displayed in the final heat maps.

## Results

### Characterization of the samples

The objective of the characterization of Syrah products and apple PA fractions described in Table 1 was elucidation of the precursors of the microbial metabolites. The apple PA fractions were the same as described in the study by Bazzocco et al. [17], describing analogous study of phenolic microbial metabolite formation from Marie Ménard and Avrolles cider apple products, as presented here. The characteristics of the apple PA fractions are shown here for comparative reasons. In the enzymatic digestion of the grape pericarp powder, the levels of PAs were not markedly affected by the digestion, although the treatment decreased the contents of anthocyanins and flavonols and increased the content of phenolic acids (Table 1). The main anthocyanins in Syrah pericarp powder were malvidin (15.94 mg/g dry powder), petunidin (3.06 mg/g dry powder) and peonidin (4.73 mg/g dry powder), as glucosides, acetyl glucosides or as *p*-coumaroyl esters. The average degree of polymerization of PAs (aDPn), describing the average number of flavanol units in the PA polymer, decreased considerably during the enzymatic digestion treatment of grapes. However, only some slight changes were observed in the carbohydrate composition of the residue: a small amount of galacturonic acid disappeared, glucose decreased slightly and fucose and galactose were slightly increased during the enzymatic treatment.

**Table 1:** Composition of Syrah grape samples before and after digestion, red wine, and grape and apple proanthocyanidin fractions

	Syrah grape		Red wine	Proanthocyanidin fractions		
	Before digestion	After digestion		Syrah	Marie Ménard (a)	Avrolles (a)
Polyphenol content mg/g dry weight						
Total proanthocyanidins	6.20	6.08	20.49	630	777	883
Total anthocyanins	25.82	9.07	5.18	n.d.	n.d.	n.d.
Total flavonols	1.06	0.49	n.d.	n.d.	n.d.	n.d.
Total phenolic acids	2.56	4.23	3.42	n.d.	n.d.	n.d.
Total polyphenols	35.64	19.87	29.09	630	777	883
Total polyphenols mg/dose (b)	3.6	2.0	2.9	16	19	22
Characteristics of proanthocyanidins						
Average degree of polymerization (aDPn)	30.72	18.76	2.84	27	9.5	35
Gallate (%)	3.4	3.5	3.54	5	N.A.	N.A.
Epigallocatechin (%)	23.02	23.35	9.55	26	N.A.	N.A.
Soluble carbohydrates						
Fructose (mg/g d.w.)	n.d.	245	n.d.	n.d.	n.d.	n.d.
Glucose (mg/g d.w.)	n.d.	168	n.d.	n.d.	n.d.	n.d.
Sucrose (mg/g d.w.)	n.d.	4	n.d.	n.d.	n.d.	n.d.
Soluble carbohydrates **(**μmol/g d.w.)	n.d.	2,318	n.d.	n.d.	n.d.	n.d.
Polymeric components (mg /g d.w.) (c)	58	66	1,000	1,000	1,000	1,000
Carbohydrate composition in polymeric components (mg/g polymers)						
Rhamnose	7	4	3	n.d.	1	n.d.
Fucose	2	17	n.d.	n.d.	n.d.	n.d.
Arabinose	40	35	12	n.d.	3	2
Xylose	17	15	2	n.d.	1	2
Mannose	4	3	14	n.d.	1	n.d.
Galactose	23	52	7	n.d.	2	1
Glucose	139	97	42	n.d.	16	18
Galacturonic acid	17	n.d.	17	n.d.	1	n.d.
Polymeric carbohydrates (μmol/g d.w.)	101	117	657	n.d.	172	153

(a) As shown in Bazzocco et al. [17]

(b) Dose 100 mg d.w. grape samples and red wine; 25 mg for proanthocyanidin fractions

(c) Polymeric components were determined as alcohol-insoluble solids corresponding to carbohydrates and flavanol polymers

Gallic acid content in the red wine was 56 g/l. The red wine contained extractable PAs that had a lower aDPn than the PAs present in pericarp powders or PA fraction (Table 1). The doses of the phenolic compounds introduced to the in vitro colon model varied, and the highest phenolic content per dose was in the PA fraction, followed by the grapes, the red wine and the digested grapes (Table 1). The red wine was also analysed after storage. The storage stability analysis (January 2006 and January 2008) showed that anthocyanins were reduced from 219 to 19 mg/l eq malvidin-3-glucoside, whereas hydroxycinnamic acids (145 and 148 mg/l eq caffeic acid, respectively) and flavanols (868 and 900 mg/l, respectively) remained rather stable. The red wine used in the fourth experiment was stored for 3 years prior to freeze-drying and used in the in vitro colon model.

### Microbial metabolism of Syrah grape products

**Fig. 2:**
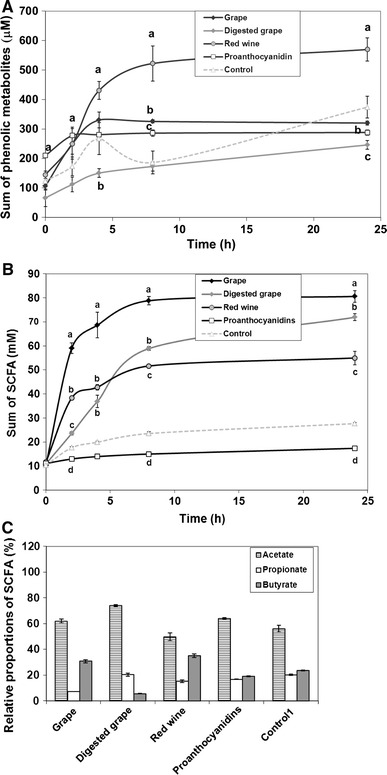
In vitro formation of **a** microbial phenolic metabolites; **b** the sum of short-chain fatty acids (SCFAs) from carbohydrates; **c** relative proportions of individual SCFAs, acetic, propionic and butyric acids formed in 24 h; from native Syrah grape pericarp powder, digested pericarp, red wine and proanthocyanidin (PA) fraction by human faecal microbiota in vitro. The sum of phenolic acid metabolites includes 3-(3′,4′-dihydroxyphenyl) propionic and -acetic acids and protocatechuic acid, 3-(3′- and 4′-hydroxyphenyl) propionic and corresponding benzoic acids, 2-(3′-hydroxyphenyl) acetic acid derivatives, and non-hydroxylated phenylpropionic and benzoic acids. The sum of SCFAs includes acetic, propionic and butyric acids. Significant (*p* < 0.05) differences from the faecal control were designated as *small letters* and *different letters* correspond to significantly (*p* < 0.05) different levels of response within a time point between substrates. The quantitated responses of the individual metabolites are collated in the Supplement Tables 2 and 3

In order to understand the effect of the sample matrix on the microbial metabolism, Syrah grape products were compared. The averages and standard deviations of the metabolite concentrations analysed by GC–MS are summarized in Supplement Table 2, and the sums of phenolic metabolites are illustrated in Fig. 2a. The screening study showed that metabolite concentrations formed by the microbiota were higher from red wine than from the grape pericarp powders or the PA fraction. The native pericarp powder with skin and pulp phenolic compounds caused a formation of moderate concentrations of the microbial metabolites. The sum of metabolites from the PA fraction or the digested berry pericarp powder did not exhibit significant difference from the faecal control, but some individual metabolites were above the control level (Supplement Table 2). The GC–MS analysis concentrated on the phenolic acids with C1-C3 side chain and with varying degrees of hydroxylation at 3′- and 4′-positions.

The degradation of grape product matrix was studied as short-chain fatty acids (SCFA) formation. The SCFA are produced from the carbohydrates from the pericarp powders or the wine matrix through the action of the faecal microbiota and from the carbohydrate residue from the dietary fibre intake of the donors in the faecal samples used in the suspension. The sum of SCFA (acetic, propionic and butyric acids; Fig. 2b) showed an increase in concentration during the incubation with faecal microbiota from the non-digested and digested pericarp powders and the red wine, whereas the PA fraction did not show SCFA formation during the incubation, which could be expected from the total lack of carbohydrates (Table 1). Surprisingly, the PA fraction showed significantly (*p* < 0.01) lower concentrations of all the SCFAs than the faecal control, indicating an inhibitory effect of the isolated, polymeric PAs on fermentation of the carbohydrates from the faecal inoculum itself by faecal microbiota. The concentrations and relative proportions of the individual SCFA are shown in Table 3 (Supplement). Acetic acid was the main SCFA in the in vitro microbial fermentation of carbohydrates. The digested pericarp powder showed the highest concentrations of propionic acid (*p* < 0.001; after 8 h), whereas the grape pericarp powders before digestion and the red wine enhanced significantly (*p* < 0.001) the butyric acid formation compared with other substrates and differed less significantly (*p* < 0.05) from each other. The digested pericarp and PA fraction showed less butyric acid formation than the faecal control (Supplement Table 3). The relative proportions of the individual SCFA after 24-h incubation are also shown in Fig. 2c.

### Comprehensive profiling of the microbial metabolites from the red wine

The red wine, showing the highest metabolite profiles in the comparison study, was chosen for the experiment with comprehensive profiling. The objective of the non-targeted comprehensive profiling of the red wine metabolites was to identify the metabolites such as tri-hydroxyphenol derivatives expected to form from epigallocatechin, gallic acid and delphinidin derivatives and methylated derivatives expected from methylated anthocyanins found in the grapes and consequently from the red wine and not analysed earlier with GC–MS. After the colon model with the freshly freeze-dried red wine, the comprehensive metabolite profiling was performed using the GCxGC-TOFMS combining targeted and non-targeted approaches. The visualization and verification of the metabolite names were performed for over-expressed metabolites after data analysis with Guineu program, as described in the Materials and Methods. The quantified targeted metabolites are shown in Table 4 (Supplement). Those metabolites that are indicated *in italics* in the text below are over-expressed metabolites showing higher responses in the chromatogram of microbial metabolites from the red wine than from the controls. The main red wine metabolites are 3-(3′-hydroxyphenyl) propionic acid, *3*-*(3′,4′*-*dihydroxyphenyl) propionic acid* and *2*-*(3′,4′*-*dihydroxyphenyl) acetic acid*. 3-Phenylpropionic acid is the most abundant metabolite; however, it is also the main compound in the faecal control (Table 4; Supplement). The minor quantified metabolites include *3*-*(4′*-*hydroxyphenyl) propionic acid (maximum 4.8* ± *0.2* μM at 8 *h, p* < *0.001),* 2-(3′-hydroxyphenyl)acetic acid (maximum 7.4 ± 0.9 μM at 2 h, *p* < 0.01), 3,4-dihydroxybenzoic acid (maximum 3.53 ± 1.20 μM, at 0 h, *p* < 0.01), 4-methylcatechol (maximum 6.4 ± 0.4 μM, at 24 h, *p* < 0.001) and vanillic acid (maximum 3.0 ± 0.7 μM, at 0 h, *p* < 0.01) (Supplement Table 4). *Gallic acid* was present at high concentration (41.7 μM, at 0 h, *p* < 0.0001) and disappeared within 2 h (0.4 ± 0.1 μM). A metabolite identified as *benzene*-*1,2,3*-*triol* could be a decarboxylation product from gallic acid (Supplement Fig. 4A). *5*-*(3′*-*Hydroxyphenyl) pentanoic acid* and *3,5*-*dimethoxy*-*4*-*hydroxybenzoic acid* were significantly over-expressed compared with the control red wine in buffer (Supplement Fig. 4B) and can structurally be connected as fragments of flavanol and malvidin precursors. Group level identifications also revealed *amino acids, carboxylic acids, phenolic acids, sugars and alcohols* showing varying fold changes in the course of time (Supplement Figs. 4A and 4B).

### Inhibitory effects of PAs on microbial conversion activities

**Fig. 3:**
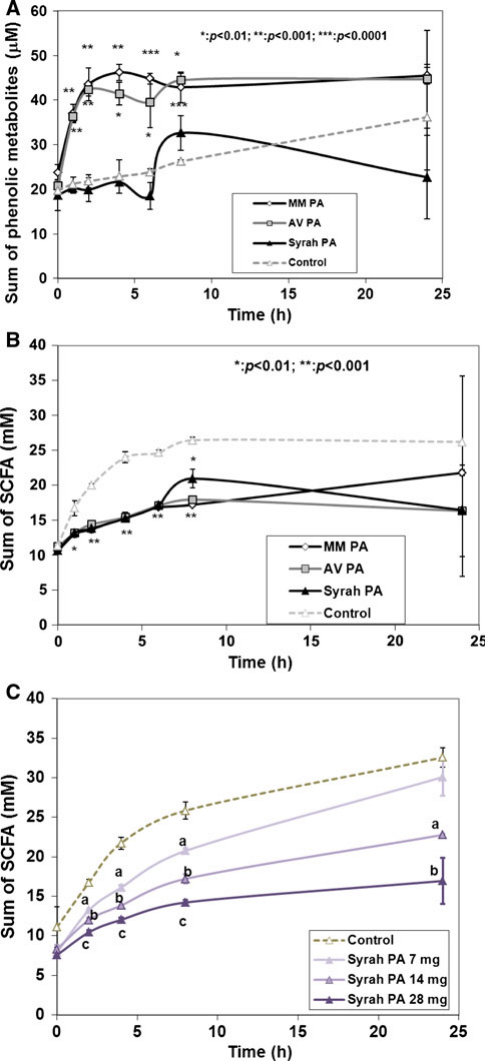
Inhibition of microbial metabolism by proanthocyanidin (PA) fractions in the in vitro colon model. MM PA: PA fraction from Marie Ménard apples; AV PA: PA fraction from Avrolles apples; Syrah PA: PA fraction from Syrah grapes. **a** The sum of phenolic metabolites includes those indicated in the caption of Fig. 2 (*p < 0.01;**p < 0.001; ***p < 0.0001). **b** The sum of SCFAs including acetic, propionic and butyric acids (*p < 0.01;**p < 0.001). *Asterisks* indicate significant difference from the control. **c** Effect of Syrah PA dose on the formation of SCFAs in the in vitro colon model by human faecal microbiota. *Letters in general* indicate significant (*p* < 0.01) differences from the control. *Different letters* indicate significant (*p* < 0.01) differences within a time point in SCFA production between doses

In order to elucidate the effect of structure of PAs on the PA-related inhibition of microbial metabolism, an additional experiment was performed including the Marie Ménard and Avrolles apple PA fractions, which were compared with the Syrah grape PA fraction. The effect of dose of the Syrah PA fraction was also tested. Fig. 3a shows that phenolic metabolites were formed from the isolated apple PAs, whereas metabolites from the grape PA fraction were at the same level as the control. Furthermore, SCFA formation was inhibited by all the PA fractions, regardless of their origin (Fig. 3b). The dose dependency was evident, because the inhibition of SCFA formation increased with the increasing dose of the Syrah PA fraction (Fig. 3c).

## Discussion

### Effects of the upper intestinal model on phenolic compounds

Usually after upper intestinal models, the digested components are removed by filtration [17] or by dialysis [50]. Unfortunately, the dialysis was not an option with samples containing proanthocyanins, because they tend to block the pores of the dialysis tubes. Thus, filtration through a cloth was used in the apple study [17], and it worked well with high-viscous apple powder containing pectic polysaccharides. However, the grape pericarp powder was liquefied during the incubation, and due to limited amount of the sample, we did not dare to perform the filtration with the cloth. Filtration would have caused the loss of phenolic compounds released from the pericarp powder and the responses would have been even lower than exhibited now. Thus, results presented here serve only as a comparison how do upper intestinal conditions affect the phenolic compounds and whether or not these changes affect the microbial conversions in the in vitro colon model.

Characterization of Syrah grape pericarp powder before and after in vitro enzymatic digestion showed changes in the phenolic composition. Grape pericarp powder was first subjected to changing pH conditions simulating the upper intestine: neutral (mouth), acidic (stomach) and neutral (duodenum) in anaerobic conditions to prevent oxidation. The concentrations of anthocyanins and flavonols decreased, the content of PAs was rather stable and phenolic acids increased after the treatment. The loss in anthocyanins was most probably due to degradation reactions in neutral pH [51]. It has been shown earlier that duodenal conditions decrease the contents of anthocyanins [52, 53]. The instability of anthocyanins in neutral or slightly basic duodenal conditions may be due to formation of colourless pseudobase from flavylium cation [51], or they may be degraded via an alpha-diketone intermediate or via hemiketal and chalcone to corresponding phenolic acids, in the case of malvidin to syringic acid (3,5-dimethoxy-4-hydroxybenzoic acid) [54].

The grape pericarp cell wall is composed of pectins (polymer containing galacturonic acid, arabinose, galactose and rhamnose), cellulose (glucose polymer) and xyloglucan hemicellulose (polymer containing xylose, non-cellulosic glucose, galactose and fucose), and they form the macromolecular pulp matrix of grapes [55]. The decrease in galacturonic acid after digestion reflected the loss of some pectic polymers. The slight increase in fucose and galactose after digestion could be due to mucin added in the enzymatic in vitro digestion. Mucin is a glycoprotein composed of amino sugars, galactose, mannose, fucose, sialic acid and uronic acids [56]. The enzymatic digestion could also make galactose and fucose polymers more susceptible for analysis, increasing their yields.

Average degree of polymerization (aDPn) of PAs in digested grape pericarp was lower than that in non-digested powder. According to Laurent, recovery of PA dimers and trimers is increased in gastric medium (depolymerization) and decreased in duodenal conditions (interaction with pancreatic proteins) [57]. The binding to proteins and enzymes is likely especially with long-chain PAs [8, 58, 59]. Neutral pH in the duodenal stage may promote oxidation [60] and enhance binding of oxidized long-chain-PAs by modification of the pericarp cell wall structure [61]. As a consequence, pH may affect solvent extraction and thiolysis in the analysis [62] and result in low aDPn values for the digested pericarp. The digestion procedure was performed under strict anaerobic conditions with a reduced buffer; however, some oxidation could have occurred immediately prior to the analysis.

### The effect of enzymatic digestion on metabolite formation

Digested grape pericarp powder showed the highest concentrations of 3-(4′-hydroxyphenyl) propionic acid. Supplement Fig. 4A showed declining fold changes (and colour intensities in the heat map) for amino acids, carboxylic acids and alcohols, suggesting decarboxylation and dehydroxylation. The transient or accumulating increase in the peak intensity shown as deeper colour in the heat maps for phenolic amino acid and phenolic acid responses (Supplement Fig. 4B) may indicate also degradation of proteins and deamination of amino acids in addition to the phenolic microbial conversions. This is supported by the transient appearance of phenolic amines and phenols (Supplement Figs. 5A and 5B), which indicate decarboxylation of phenolic amino acids and possible deamination. The decline of phenols is likely to occur via dehydroxylation, which is very common in the metabolism of phenolic compounds [63]. In the colon model, an increase in 3-(4′-hydroxyphenyl) propionic acid from the digested grape may thus be an indication of degradation of proteins from alimentary enzymes rather than a token of conversion of phenolic compounds, explaining the difference between the colonic degradation of digested and non-digested grapes and low accumulation of other phenolic microbial metabolites from the digested pericarp powder.

 The concentration of 3-phenylpropionic acid was also high in the controls, indicating other precursors originating from the diets of the donors of the faeces rather than from grape phenolic compounds. Digested pericarp powder showed lower concentrations of this metabolite than from the faecal control, whereas 3-phenylpropionic acid concentration from red wine was above the control.

### Comparison of microbial metabolite formation from Syrah grape products

The product comparison of colonic microbial metabolites in vitro revealed differences between the non-digested and the digested grape pericarp powders and the red wine. The red wine showed the highest sum of phenolic metabolites compared with those from pericarp powders, even though the red wine dose was lower than that of the pericarp powder before digestion. Lower concentration of the low aDPn PAs and other monomeric phenolics in the red wine caused the highest concentrations of the metabolites, indicating that conversion is more dependent on the chain length of the PAs than the concentration of the precursors. The aDPn of PAs in the Syrah products varied, and we have shown earlier that the susceptibility of PAs to actions of faecal microbiota in vitro is dependent on their chain length [17]. The total yields of metabolites decrease significantly with increasing polymerization [17, 64, 65]. Even catechin dimers showed highest metabolite concentrations at later time points than catechin monomers, indicating a slower conversion rate [16, 19]. Proanthocyanidins have shown to have a low convertability by human microbiota, as shown by Deprez et al. [66] when only 9–22 % of the label from [^14^C]-PA was in the metabolite pool after in vitro conversion with human microbiota. Long-chain PAs are poorly extractable into the wine [67], explaining the low aDPn in red wine samples. Solubility of low aDPn PAs and hydroxycinnamic acids makes the red wine phenolic compounds easily accessible to the microbiota for conversions. On the other hand, high doses of PAs with high aDPn in the PA fraction were able to inhibit the conversions. The earlier maxima and low conversion efficiency shown by Bazzocco and co-workers [17] for apple products suggests that there was possibly not time enough to finish the conversions.

The concentrations of microbial benzoic acid metabolites were not detected in the comparison of the Syrah products in the first experiment. The formation of such metabolites as 3′,4′,5′-trihydroxyphenyl and 3′,5′-dimethoxy-4′-hydroxyphenyl derivatives, which could be formed from epigallocatechin units, anthocyanins and gallic acid, were not monitored in the GC–MS analysis at this stage. Motivated by this gap in the analysis, the continuation of the study was conducted with more comprehensive metabolic profiling, enabling the use of more standards and revealing complementary, previously unknown metabolites.

### Profiling of microbial metabolites from red wine

In our red wine microbial metabolite profile (GCxGCTOFMS), we found the following possible metabolites: 3-hydroxyphenyl pentanoic acid (5-(3′-hydroxyphenyl) valeric acid) and corresponding phenylpropionic and phenylacetic acids. A number of studies support our findings: Deprez et al. and Tourino and co-workers demonstrated their formation from a purified PA polymer in an in vitro incubation with human microbiota and in rat urine after administration of grape dietary fibre, respectively [66, 68]. When (−)-epicatechin dimers were incubated with human colonic microbiota, the main metabolites were 2-(3′,4′-dihydroxyphenyl) acetic acid and 5-(3′,4′-dihydroxyphenyl)-γ-valerolactone [16]. Valerolactone derivatives were not identified in our study, because standards were not commercially available. Catechin isomers and their gallates incubated in pig *caecal* microbiota in vitro showed phenylpropionic acids, -acetic acids, benzoic acid derivatives and phloroglucinol [69]. Stoupi and co-workers reported that phenylacetic acid derivatives were produced from the upper unit; valerolactones, phenylvaleric acid derivatives and hydroxyphenyl hydracrylic acid originated from the lower unit of the dimer [70, 71]. Furthermore, (+)-catechin and (−)-epicatechin were precursors of phenylpropionic acids or -valeric acids depending on the inoculum and its donors and not on the stereoisomerism [19]. These earlier findings are in accordance with our current study despite the different origins of the microbiota.

The targeted analytical approach verifies the findings obtained by the non-targeted metabolite profiling. The dominating metabolites in red wine incubation analysed by targeted GCxGC-TOFMS were 3-(3′-hydroxyphenyl) propionic acid, 2-(3′,4′-hydroxyphenyl) acetic acid and benzoic acid, but 3-phenylpropionic acid was also significantly above the control, suggesting complete dehydroxylation of 3-(3′-hydroxyphenyl) propionic acid. Furthermore, galloylated catechins epigallocatechin units (comparison of 26 % of PA; with a 3′,4′,5′-trihydroxylated B-ring), epicatechin gallate (5 % of the PA) and free gallic acid could be precursors of the analysed benzoic acid metabolites formed via ring fission or dehydroxylation reactions.

The structures of the minor metabolites formed from the red wine reflect also the structures of anthocyanins present in Syrah grapes: Vanillic acid (3-methoxy-4-hydroxy benzoic acid), protocatechuic acid (3,4-dihydroxybenzoic acid) and syringic acid (3,5-dimethoxy-4-hydroxybenzoic acid) have structural resemblance to the main Syrah anthocyanins: malvidin (with a 3′,5′-dimethoxy-4′-hydroxy B-ring), petunidin (with a 3′,4′-dihydroxy-5′-methoxy B-ring) and peonidin (with a 3′-methoxy-4′-hydroxy B-ring). These minor metabolites could originate from their anthocyanin parent compound. The connection between anthocyanin precursors and microbial benzoic acid metabolites has been described earlier by several authors [18, 54, 72, 73]. However, Fleschhut and co-workers [54] demonstrated that anthocyanin degradation occurs easily also spontaneously prior to the microbial metabolism in the upper intestine (neutral conditions), and thus, spontaneous degradation of anthocyanins during the storage of the red wine is also highly likely, which was supported in our study by the early maxima of 3,4-dihydroxybenzoic acid and vanillic acid, and highest fold-change of 3,4-dimethoxy-4-hydroxybenzoic acid at 0 h time point in the heat map (Supplement Fig. 4B). Thus, the formation of benzoic acid derivatives is more likely due to non-microbial degradation of anthocyanins than caused by faecal microbial conversion reactions in this study.

Hydroxycinnamic acids could also contribute to the 3-(3′-hydroxyphenyl) propionic acid and benzoic acid concentrations via reduction in the double bond and by shortening of the side chain by two carbons (β-oxidation), as reported for caffeic acids and their caftaric esters [20], the latter being abundant in red wine. 4-Methylcatechol, a minor metabolite in this study, may also be formed from caffeic acid via shortening of the side chain by one carbon unit (α-oxidation) and loss of acid group (decarboxylation) [74]. Furthermore, faecal microbiota can easily degrade methoxyl- and hydroxyl-groups [63, 75, 76], which is also apparent in this study, for example, as declining fold changes of 3,5-dimethoxy-4-hydroxybenzoic acid (Supplement Fig. 4B) and decreasing concentrations of vanillic acid (Supplement Table 4).

### Suppression of phenolic microbial metabolite and SCFA formation by PA fractions

Isolated PA fractions from different fruits (Marie Ménard and Averolles apples and Syrah grapes) were compared for their ability to suppress microbial metabolism to clarify, if the inhibition were dependent on the chain length or origin of PAs. Suppression of the formation of the phenolic metabolites was less pronounced for apple PAs than for Syrah PA fraction. Some phenolic metabolites were formed in the presence of apple PAs. The sum of phenolic metabolites was lowest from the Syrah grape fraction.

The characteristics of the PA fractions used in this study indicate that the Syrah PA fraction had an aDPn of 27 with 5 % galloylated units and 26 % epigallocatechin units, whereas Marié Ménard apple PA fraction contained more shorter chains (aDPn 9.5) than Avrolles PA fraction (aDPn 35) [17]. This is in agreement with the results of Souquet et al. [2] and Sanoner et al. [77]. Longer chain PAs extracted from Avrolles variety exhibited a slightly stronger suppression of phenolic acid metabolism than Marie Ménard PA fraction, as shown earlier by Bazzocco et al. [17]. Although Avrolles PA and Syrah PA fractions both have high aDPn, they still had clearly different capacities of suppression of the phenolic metabolism, the sum of the metabolites from the Syrah PA being at the level of that from the faecal control. A part of the suppression could be due to actual inhibition of faecal enzymes in the presence of more galloylated Syrah PAs, but it could partly be due to the lack of standards of the specific benzoic acid derivatives, derived from epigallocatechin units and gallic acid. Consequently, the comparison of PA fractions could show a lower curve for Syrah metabolites than those for the apple PA fractions, exhibiting more metabolites from flavonoid precursors. However, the suppression of SCFA acid supports the inhibitory effect.

Formation of SCFA, as the control, is caused by the faecal inoculum containing remnants of non-fermented carbohydrates from the diet of the donors, which serves as a carbohydrate source for the SCFA background curve [48]. This background SCFA formation should have been shown in the presence of PA fractions, but all the PA fractions strongly inhibited SCFA formation independently of their origin, and this was dose dependent, as shown for Syrah PAs.

Suppression of microbial metabolites in the in vitro colon model can be caused by enzymatic inhibition, when the inhibiting compound is bound to the active site of the enzyme and its substrate cannot be attached to it. The differences in the inhibition between Syrah and apple PAs could also be due to (−)-epicatechin gallate molecules in grape, which has a well-exposed galloyl moiety enabling more efficient binding to several sites in the proteins and the formation of complexes with metal ions [8]. The binding increases with the degree of polymerization [15] and with the degree of galloylation [8]. This may well contribute to the strong, inhibition of Syrah fraction reported in the present study. Zdunczyk et al. [78] observed inhibition of caecal bacterial β-glucosidase, and α- and β-galactosidase in rats fed with grapefruit polyphenol extract. Soares and co-workers showed that pectin formed a complex with α-amylase and PA [79]. Levrat et al. demonstrated inhibition of SCFA formation from pectin in rats in the presence of pre-fermented condensed tannins (Quebracho), which were still degraded to polar phenolic compounds [80]. This is supporting our presented results of stronger inhibition of SCFA formation than that of the phenolic microbial metabolites.

The inhibition of SCFA formation occurred in the absence of the fruit or beverage matrix in this study and in that of Bazzocco et al. [17]. Lack of inhibition by grape and apple PAs within product matrices could be due to the absence of interactive, free hydroxyl moieties of PAs, which were occupied by pectin and protein interactions. Especially, in the presence of the tannin-rich Syrah red wine, the short PAs were able to suppress neither phenolic microbial metabolite nor SCFA formation, even though they are known to bind to proteins and cause astringency, a feel of dry mouth when tannin-rich wine is tasted [11]. Tannins in feeds have caused anti-nutritional effects by forming complexes with proteins in the feeds [81]. The dose of PA fractions was high enough to inhibit enzymes [81, 82] and to show the almost complete suppression of formation of SCFA and a slight suppression of the microbial phenolic metabolites.

## Conclusions

The current study aimed at revealing factors affecting the intestinal microbial conversions of polyphenols from Syrah grape products and further to elucidate the microbial metabolite profile formed from the Syrah red wine in the in vitro colon model. The factors affecting the formation of the microbial phenolic metabolites and SCFA were related to PA structure, chain length and diversity of metabolites derived from galloylated Syrah catechins. The metabolite formation was strongly affected also by the presence of the fruit or beverage matrix, suggesting that the isolation of PAs from the matrix causes luminal interactions with enzymes and proteins, but consumption of PA-rich fruits and beverages does not. Furthermore, the diversity of metabolites could be elucidated by the non-targeted approach using the comprehensive metabolite profiling, which can provide non-selective data sets for data analysis and identification, not limited by the existing standards. Metabolite profiling was complementary to the targeted analysis. The identified metabolites had biological relevance, because the structures of the metabolites resembled fragments of their grape phenolic precursors or were in agreement with literature data.

## Electronic supplementary material

Below is the link to the electronic supplementary material.

Supplementary material 1 (DOCX 243 kb)

## References

[1] Prieur C, Rigaud J, Cheynier V, Moutounet M (1994). Oligomeric and polymeric procyanidins from grape seeds. Phytochemistry.

[2] Souquet J-M, Cheynier V, Brossaud F, Moutounet M (1996). Polymeric proanthocyanidins from grape skins. Phytochemistry.

[3] Mané C, Souquet JM, Ollé D, Verriés C, Véran F, Mazerolles G, Cheynier V, Fulcrand H (2007). Optimization of simultaneous flavanol phenolic acid, and anthocyanin extraction from grapes using an experimental design: application to the characterization of champagne grape varieties. J Agric Food Chem.

[4] Mattivi F, Guzzon R, Vrhovsek U, Stafanini M, Velasco R (2006). Metabolite profiling of grape: flavonols and anthocyanins. J Agric Food Chem.

[5] Adrian M, Jeandet P, Douillet-Breuil AC, Bessis R (2000). Stilbene content of mature *Vitis vinifera* berries in response to UV-C elicitation. J Agric Food Chem.

[6] Fournand D, Vicens A, Sidhoum L, Souquet J-M, Moutonet M, Cheynier V (2006). Accumulation and extractability of grape skin tannins and anthocyanins at different advanced physiological stages. J Agric Food Chem.

[7] Somers TC (1971). The polymeric nature of wine pigments. Phytochemistry.

[8] Le Bourvellec C, Renard CMGC (2012). Interactions between polyphenols and macromolecules: quantification methods and mechanisms. Crit Rev Food Sci Nutr.

[9] Sacchi LK, Bisson LF, Adams DO (2005). A review of the effect of winemaking techniques on phenolic extraction in red wines. Am J Enol Vitic.

[10] Morel-Salmi C, Souquet JM, Bes M, Cheynier V (2006). The effect of flash release treatment on phenolic extraction and wine composition. J Agric Food Chem.

[11] Cheynier V, Prieur C, Guyot S, Rigaud J, Moutounet M (1997) The structures of tannins in grapes and wines and their interactions with proteins. In: Watkins T (ed) A.C.S. symposium. Series wine; nutritional and therapeutic benefits, pp 81–93

[12] Canals R, Llaudy MC, Valls J, Canals JJ, Zamora F (2006). Influence of ethanol concentration on the extraction of color and phenolic compounds from the skins and seeds of Tempranillo grapes at different stages of ripening. J Agric Food Chem.

[13] Donovan JL, Manach C, Rios L, Morand C, Scalbert A, Rémésy C (2002). Procyanidins are not bioavailable in rats fed a single meal containing a grapeseed extract of the procyanidin dimer B_3_. Br J Nutr.

[14] Haslam E (1996). Natural polyphenols (vegetable tannins) as drugs: possible models of action. J Nat Prod.

[15] Scalbert A (1991). Antimicrobial properties of tannins. Phytochemistry.

[16] Appeldoorn MM, Vincken J-P, Aura A-M, Hollman PCH, Gruppen H (2009). Procyanidin dimers are metabolised by human microbiota with 2-(3,4-dihydroxyphenyl)-acetic acid and 5-(3,4-dihydroxyphenyl)-gamma-valerolactone as the major metabolites. J Agric Food Chem.

[17] Bazzocco S, Mattila I, Guyot S, Renard CMGC, Aura A-M (2008). Factors affecting the conversion of apple polyphenols to phenolic acids and fruit matrix to short-chain fatty acids by human faecal microbiota in vitro. Eur J Nutr.

[18] Aura A-M, Martin-Lopez P, O′Leary KA, Williamson G, Oksman-Caldentey K, Poutanen K, Santos-Buelga C (2005). In vitro metabolism of anthocyanins by human gut microflora. Eur J Nutr.

[19] Aura A-M, Mattila I, Seppänen-Laakso T, Miettinen J, Oksman-Caldentey K-M, Orešič M (2008). Microbial metabolism of catechin stereoisomers by human faecal microbiota: comparison of targeted analysis and a non-targeted metabolomics method. Phytochem Lett.

[20] Gonthier M, Rémésy C, Scalbert A, Cheynier V, Souquet J-M, Poutanen K, Aura A-M (2006). Microbial metabolism of caffeic acid and its esters chlorogenic and caftaric acids by human faecal microbiota in vitro. Biomed Pharmacotherapy.

[21] Auger C, Al-Awwadi N, Bornet A, Rouanet J-M, Gasc F, Cros G, Teissedre P-L (2004). Catechins and procyanidins in Mediterranean diets. Food Res Int.

[22] Ovaskainen M-L, Törrönen R, Koponen JM, Sinkko H, Hellström J, Reinivuo H, Mattila P (2008). Dietary intake and major food sources of polyphenols in finnish adults. J Nutr.

[23] World Health Organization (2003) Diet, nutrition and the prevention of chronic diseases. WHO Technical Report Series, pp 1–149 12768890

[24] Grassi D, Desideri G, Necozione S, Lippi C, Casale R, Properzi G, Blumberg JB, Ferri C (2008). Blood pressure reduced and insulin sensitivity increased in glucose intolerant, hypertensive subjects after 15 days of consuming of high-polyphenol dark chocolate. J Nutr.

[25] Tomaru M, Takano H, Osakabe N, Yasuda A, Inoue K, Yanagisawa R, Ohwatari T, Uematsu H (2007). Dietary supplementation with cacao liquor proanthocyanidins prevents elevation of blood glucose levels in diabetic obese mice. Nutrition.

[26] Lee YA, Cho EJ, Yokozawa T (2008). Effects of proanthocyanidin preparations on hyperlipidemia and other biomarkers in mouse model of type 2 diabetes. J Agric Food Chem.

[27] Pinent M, Bladé C, Salvadó MJ, Blay M, Pujadas G, Fernández-Larrea J, Arola L, Ardévol A (2006). Procyanidin effects on adipocyte-related pathologies. Crit Rev Food Sci Nutr.

[28] Adisakwattana S, Moonsan P, Yibchok-anun S (2008). Insulin-releasing properties of a series of cinnamic acid derivatives in vitro and in vivo. J Agric Food Chem.

[29] Jayaprakasam B, Vareed SK, Olson SK, Nair MG (2005). Insulin secretion by bioactive anthocyanins and anthocyanidins present in fruits. J Agric Food Chem.

[30] Bäckhed F, Manchester JK, Semenkovich CF, Gordon JI (2007). Mechanisms underlying the resistance to diet induced obesity in germ-free mice. Proc Natl Acad Sci USA.

[31] Robertson MD (2007). Metabolic cross talk between the colon and the periphery: implications for insulin sensitivity. Proc Nutr Soc.

[32] Sawai Y, Kohsaka K, Nishiyama Y, Ando K (1987). Serum concentrations of rutoside metabolites after oral administration of a rutoside formulation to humans. Drug Res.

[33] Gross M, Pfeiffer M, Martini M, Campbell D, Slavin J, Potter J (1996). The quantitation of metabolites of quercetin flavonols in human urine. Cancer Epidemiol Biomark Prev.

[34] Kuijsten A, Arts ICW, Vree TB, Hollman PCH (2005). Pharmacokinetics of enterolignans in healthy men and women consuming a single dose of secoisolariciresinol diglucoside. J Nutr.

[35] Katajamaa M, Orešič M (2007). Data processing for mass spectrometry-based metabolomics. J Chromatography A.

[36] Castillo S, Mattila I, Miettinen J, Orešič M, Hyötyläinen T (2011). Data analysis tool for comprehensive two-dimensional gas chromatography-time of flight mass spectrometry. Anal Chem.

[37] Guyot S, Marnet N, Drilleau JF (2001). Thiolysis-HPLC characterization of apple procyanidins covering a large range of polymerization states. J Agric Food Chem.

[38] Kennedy J, Jones GP (2001). Analysis of proanthocyanidin cleveage products following acid-catalysis in the presence of excess phloroglucinol. J Agric Food Chem.

[39] Ducasse M-, Canal-Llauberes R-, de Lumley M, Williams P, Souquet J-M, Fulcrand H, Doco T, Cheynier V (2010). Effect of macerating enzyme treatment on the polyphenol and polysaccharide composition of red wines. Food Chem.

[40] Guyot S, Marnet M, Sanoner P, Drilleau J-F (2001). Direct thiolysis on crude apple materials for high-performance liquid chromatography characterization and quantification of polyphenols in cider apple tissues and juices. Methods Enzymol - Flavonoïds and other Polyphenols.

[41] Renard CMGC (2005). Variability in cell wall preparations: quantification and comparison of common methods. Carbohydr Polym.

[42] Englyst HN, Cummings JH (1984). Simplified method for the measurement of total non-starch polysaccharides by gas-liquid chromatography of constituent sugars as alditol acetates. Analyst.

[43] Seaman JF, Moore WE, Mitchell RL, Millett MA (1954). Techniques for the determination of pulp constituents by quantitative paper chromatography. TAPPI.

[44] Blumenkrantz N, Asboe-Hansen G (1973). New method for quantitative determination of uronic acids. Anal Biochem.

[45] Aura A-M, Härkönen H, Fabritius M, Poutanen K (1999). Development of an in vitro enzymic digestion method for removal of starch and protein and assessment of its performance using rye and wheat breads. J Cereal Sci.

[46] Aura A-M, O’Leary KA, Williamson G, Ojala M, Bailey M, Puupponen-Pimiä R, Nuutila AM, Oksman-Caldentey K, Poutanen K (2002). Quercetin derivatives are deconjugated and converted to hydroxyphenylacetic acids but not methylated by human fecal flora in vitro. J Agric Food Chem.

[47] Durand M, Dumay C, Beaumatin P, Morel MT (1988). Use of rumen simulation technique (RUSITEC) to compare microbial digestion of various by-products. Animal Feed Sci Tech.

[48] Aura A-M, Oikarinen S, Mutanen M, Heinonen S, Adlercreutz HCT, Virtanen H, Poutanen KS (2006). Suitability of a batch in vitro fermentation model using human faecal microbiota for prediction of conversion of flaxseed lignans to enterolactone with reference to an in vivo rat model. Eur J Nutr.

[49] Aura A-M, Mattila I, Hyötyläinen T, Gopalacharyulu P, Bounsaythip C, Oresic M, Oksman-Caldentey K-M (2011). Drug metabolome of the Simvastatin formed bu human intestina microbiota in vitro. Mol BioSyst.

[50] Karppinen S, Liukkonen K, Aura A-M, Forssell P, Poutanen K (2000). In vitro fermentation of polysaccharides of rye, wheat and oat brans and inulin by human faecal bacteria. J Sci Food Agric.

[51] Clifford MN (2000). Anthocyanins—nature, occurrence and dietary burden. J Sci Food Agric.

[52] Tagliazucchi D, Verzelloni E, Bertolini D, Conte A (2010). In vitro bioaccessibility and anti-oxidant activity of grape polyphenols. Food Chem.

[53] Bouayed J, Hoffman L, Bohn T (2011). Total phenolic¸ flavonoids, anthocyanins and antioxidant activity following simulated gastro-intestinal digestion and dialysis of apple varieties: bioaccessibility and potential uptake. Food Chem.

[54] Fleschhut J, Kratzer F, Rechkemmer G, Kulling SE (2006). Stability and biotransformation of various dietary anthocyanins in vitro. Eur J Nutr.

[55] Vidal S, Williams P, O’Neill MA, Pellerin P (2001). Polysaccharides from grape berry cell walls. Part I: tissue distribution and structural characterization of the pectic polysaccharides. Carb Polym.

[56] Killer J, Marounek M (2011). Fermentation of mucin by bifidobacteria from rectal samples of humans and rectal and intestinal samples of animals. Folia Microbiol.

[57] Laurent C, Besancon P, Caporiccio B (2007). Flavonoids from a grape seed extract with digestive secretion and intestinal cells as assessed in an in vitro digestion/Caco-2 cell culture model. Food Chem.

[58] Sarni-Manchado P, Deleris A, Avalone S, Cheynier V, Moutounet M (1999). Analysis and characterization of wine condensed tannins precipitated by protein used as fining agent in enology. Am J Enol Vitic.

[59] Maury C, Sarni-Manchado P, Lefebvre S, Cheynier V, Moutounet M (2001). Influence of fining with different molecular weight gelatins on proanthocyanidin composition and perception of wines. Am J Enol Vitic.

[60] Poncet-Legrand C, Cabane B, Bautista-Ortin AB, Carrillo S, Fulcrand H, Perez J, Vernhet A (2010). Tannin oxidation: infra- versus intermolecular reactions. Biomacromolecules.

[61] Le Bourvellec C, Guyot S, Renard CMGC (2009). Interactions between apple (Malus × domestica Borkh.) polyphenols and cell walls modulate the extractability of polysaccharides. Carbohydr Polym.

[62] Guyot S, Marnet N, Sanoner P, Drilleau JF (2003). Variability of the polyphenolic composition of cider apple (*Malus domestica*) fruits and juices. J Agric Food Chem.

[63] Rechner AR, Smith MA, Kuhnle G, Gibson GR, Debham ES, Srai SKS, Moore KP, Rice-Evans CA (2004). Colonic metabolism of dietary polyphenols: influence of structure on microbial fermentation products. Free Rad Biol Med.

[64] Gonthier M-P, Donovan JL, Texier O, Felgines C, Rémésy C, Scalbert A (2003). Metabolism of dietary procyanidins in rats. Free Rad Biol Med.

[65] Rios LY, Gonthier M-P, Rémesy C, Mila I, Lapierre C, Lazarus SA, Williamson G, Scalbert A (2003). Chocolate intake increases urinary excretion of polyphenol-derived phenolic acids in healthy human subjects. Am J Clin Nutr.

[66] Deprez S, Brezillon C, Rabot S, Phillippe C, Mila I, Lapierre C, Scalbert A (2000). Polymeric proanthocyanidins are catabolized by human colonic microflora into low-molecular-weight phenolic acids. J Nutr.

[67] Cheynier V, Duenas-Paton M, Salas E, Maury C, Souquet J-, Sarni-Manchado P, Fulcrand H (2006). Structure and properties of wine pigments and tannins. Am J Enol Vitic.

[68] Tourino S, Fuguet E, Vinardell MP, Cascante M, Torres JL (2009). Phenolic metabolites of grape antioxidant dietary fibre in rat urine. J Agric Food Chem.

[69] Slot van′t G, Humpf H (2009). Degradation and metabolism of catechin, epicatechin-3-gallate (EGCG), and related compounds by the intestinal microbiota in the pig cecum model. J Nutr.

[70] Stoupi S, Williamson G, Drynan JW, Barron D, Clifford MN (2010). Procyanidin B2 catabolism by human fecal microflora: partial characterization of ‘dimeric’ intermediates. Arch Biochem Biophys.

[71] Stoupi S, Williamson G, Drynan JW, Barron D, Clifford MN (2010). A comparison of the in vitro transformation of (−)-epicatechin and proanthocyanidin B2 by human faecal microbiota. Mol Nutr Food Res.

[72] Forrester SC, Waterhouse AL (2008). Identification of cabernet sauvignon anthocyanin gut microflora metabolites. J Agric Food Chem.

[73] Keppler K, Humpf HU (2005). Metabolism of anthocyanins and their phenolic degradation products by the intestinal microflora. Bioorg Med Chem.

[74] Peppercorn MA, Goldman P (1971). Caffeic acid metabolism by bacteria of the human gastrointestinal tract. J Bacteriol.

[75] Blaut M, Clavel T (2007). Metabolic diversity of the intestinal microbiota: implications for health and disease. J Nutr.

[76] Aura A-M (2008). Microbial metabolism of dietary phenolic compounds in the colon. Phytochem Rev.

[77] Sanoner P, Guyot S, Marnet N, Molle D, Drilleau JF (1999). Polyphenol profiles of French cider apple varieties (*Malus domestica* sp.). J Agric Food Chem.

[78] Zdunczyk Z, Juskievicz J, Estrella I (2006). Cecal parameters of rats fed diets containing grapefruit polyphenols and inulin as single supplements or in a combination. Nutrition.

[79] Soares SI, Goncalves RM, Mateus N, de Freitas V (2009). Mechanistic approach by which polysaccharides inhibit α-amylase/procyanidin aggregation. J Agric Food Chem.

[80] Levrat M-A, Texier MDO, Régerat F, Demingé C, Rémésy C (1993). Comparison of the effects of condensed tannin and pectin cecal fermentations and lipid metabolism in the rat. Nutr Res.

[81] Goel G, Puniya AK, Aguilar CN, Singh K (2005). Interaction of gut microflora with tannins in feeds. Naturwissenschaften.

[82] Le Bourvellec C, Le Quéré JM, Sanoner P, Guyot S, Drilleau JF (2004). Inhibition of apple polyphenol oxidase activity by procyanidins and polyphenol oxidation products. J Agric Food Chem.

