# Supplementary material for: Characterization of microbial metabolism of Syrah grape products in an in vitro colon model using targeted and non-targeted analytical approaches
Source: Eur J Nutr. 2012 Jun 16;52(2):833–46. doi: 10.1007/s00394-012-0391-8 (PMC3573183; doi:10.1007/s00394-012-0391-8)

Supplement to

**Characterization of microbial metabolism of Syrah grape products in an *in vitro* colon model using targeted and non-targeted analytical approaches**

# Anna-Marja Aura^1^*, Ismo Mattila^1^, Tuulia Hyötyläinen^1^, Peddinti Gopalacharyulu^1^, Veronique Cheynier^2^, Jean-Marc Souquet^2^, Magali Bes^3^, Carine Le Bourvellec^4^, Sylvain Guyot^5^ and Matej Orešič^1^

^1^* VTT Technical Research Centre of Finland, P.O.Box 1000, Tietotie 2, Espoo, FI-02044 VTT, Finland Tel: + 358 20 722 6178; Fax: + 358 20 722 7071; E-mail: anna-marja.aura@vtt.fi

^2^ INRA, UMR 1083, Sciences Pour l'œnologie, Montpellier, France

^3^ INRA, Unité Expérimentale de Pech Rouge, 11 430 Gruissan, France

^4^ INRA, UMR 408 "Sécurité et Qualité des Produits d'Origine Végétale", Avignon, France

^5^ INRA UR117 Cidricoles et Biotransformation des Fruits et Légumes. Le Rheu, France

Tables 2-4

***Fig.4.* Heat maps of over-expressed microbial metabolites from Syrah red wine by human faecal microbiota against controls: A. Microbiota without added red wine (Faecal control); B. Red wine without microbiota (Red wine in buffer). The asterisks shown in the heat map indicate significance of difference in means at each time point based on the t-test (*:p<0.05; **:p<0.01; ***p<0.001). The quantitated responses of the phenolic microbial metabolites are collated in the Supplement Table 4.**

**Fig. 5A. Time course decline of alcohols, amino acids and carboxylic acids during *in vitro* incubation with human faecal microbiota.**


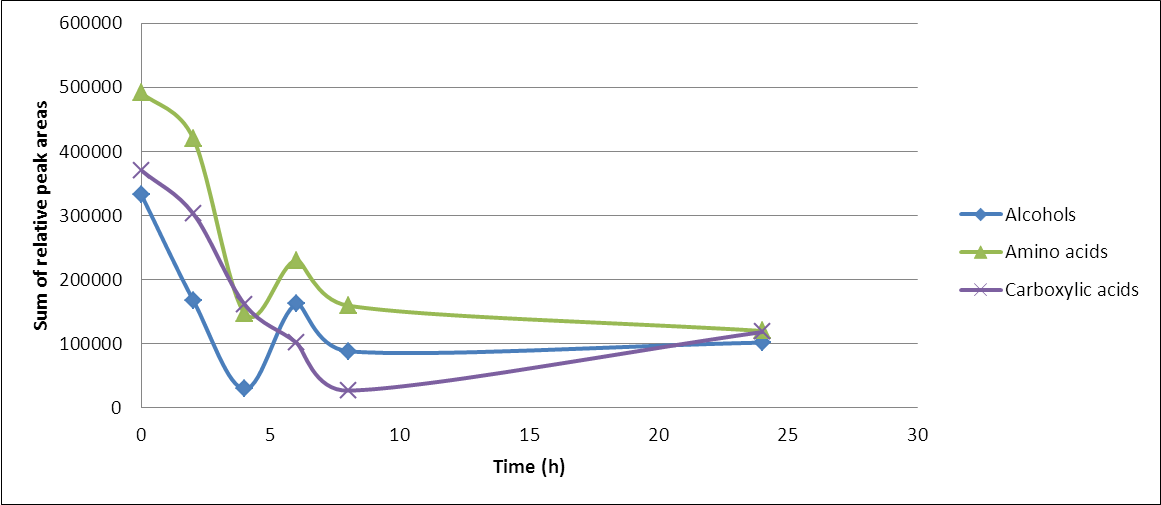


**Fig. 5B. Time course decline of amines, phenols, phenolic acids, phenolic amines and phenolic amino acids during *in vitro* incubation with human faecal microbiota.**


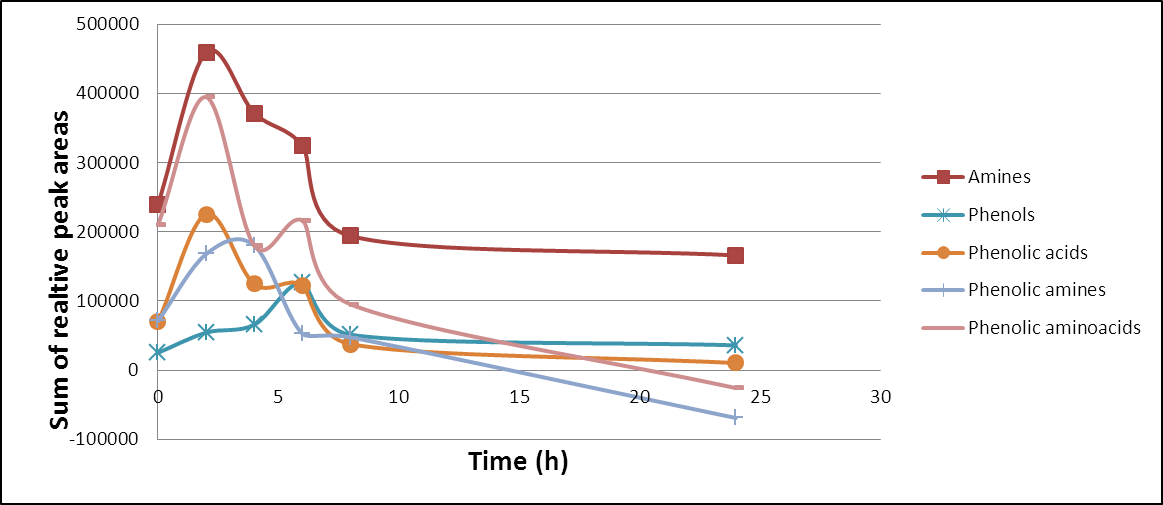

Supplement: Supplementary file 1 — Supplementary material 1 (DOCX 243 kb) [file 394_2012_391_MOESM1_ESM.docx]
